# Identification and characterization of microsatellites in *Aconitum reclinatum* (Ranunculaceae), a rare species endemic to North America

**DOI:** 10.1002/aps3.1161

**Published:** 2018-06-24

**Authors:** Juan Zhou, Wanzhen Liu, Hanghui Kong, Wei Gong

**Affiliations:** ^1^ College of Life Sciences South China Agricultural University Guangzhou 510614 People's Republic of China; ^2^ Key Laboratory of Plant Resources Conservation and Sustainable Utilization South China Botanical Garden Chinese Academy of Sciences Guangzhou 510650 People's Republic of China; ^3^ Guangdong Provincial Key Laboratory of Applied Botany South China Botanical Garden Chinese Academy of Sciences Guangzhou 510650 People's Republic of China

**Keywords:** *Aconitum reclinatum*, endangered species, herbal medicine, microsatellites, next‐generation sequencing, Ranunculaceae

## Abstract

**Premise of the Study:**

*Aconitum reclinatum* is the only representative species of *Aconitum* subg. *Lycoctonum* in North America, with restricted ranges and endangered populations. Polymorphic microsatellite markers were developed for *A. reclinatum* for further investigation of genetic diversity and population structure.

**Methods and Results:**

Using Illumina HiSeq technology, we sequenced a genomic library for identification of simple sequence repeat markers. A total of 12 polymorphic primer pairs were developed and tested on 66 individuals from four populations in North America. The number of alleles ranged from one to seven per locus with an average of 3.48. Levels of observed and expected heterozygosity varied from 0 to 1.000 and 0 to 0.736, respectively, at population level. Three primer pairs were successfully amplified in three of four closely related species.

**Conclusions:**

The microsatellites isolated in this study will be useful in further research on the genetic diversity and conservation genetics of *A. reclinatum* populations in North America.

*Aconitum reclinatum* A. Gray, also known as white monkshood, is a perennial herb belonging to *Aconitum* L. subg. *Lycoctonum* (DC.) Peterm. in Ranunculaceae. *Aconitum* subg. *Lycoctonum* is composed of species distributed throughout Eurasia and North America (Jabbour and Renner, 2012). *Aconitum reclinatum* is the only representative species of this subgenus in North America, where it has a restricted geographic range. The largest existing populations of *A. reclinatum* are known to be located in North Carolina in the eastern United States (Hardin, 1964; Brink, 1982). This species is reportedly disturbed and threatened by logging activities and drainage of its habitat (NatureServe, 2007). To date, the genetic diversity and population genetic structure are still unclear for *A. reclinatum* populations.

Although some species in the genus *Aconitum* are highly toxic because of aconite alkaloids, it is popularly used as a traditional herbal medicine in Asia (Liang et al., 2017). Previous research has been conducted to reveal the genetic variation among some populations in *Aconitum*, using traditional molecular markers including amplified fragment length polymorphism (AFLP), inter‐simple sequence repeat (ISSR), and random‐amplified polymorphic DNA (RAPD) markers (Cole and Kuchenreuther, 2001; Meng et al., 2014; Zhao et al., 2015). Most recently, microsatellites have been developed and characterized for some *Aconitum* species based on next‐generation sequencing (He et al., 2015; Ge et al., 2016). However, those microsatellite markers cannot be amplified in *A. reclinatum* successfully due to low interspecific transferability. Therefore, we developed microsatellite markers specific for *A. reclinatum* for further application in the investigation of genetic diversity and population structure. A total of 12 polymorphic microsatellites were isolated and characterized, which will provide information to interpret the fine population structure of this rare and threatened species.

## METHODS AND RESULTS

Tender leaves were collected from *A. reclinatum* and dried instantly in silica gel. A total of 66 individuals and four populations were sampled. Total genomic DNA was extracted from the dried leaves of *A. reclinatum* using a modified cetyltrimethylammonium bromide (CTAB) method (Doyle and Doyle, 1987). The DNA concentration was quantified using a NanoDrop spectrophotometer (Thermo Fisher Scientific, Carlsbad, California, USA), and a final DNA concentration of >30 ng/μL was used subsequently.

We used one individual of *A. reclinatum* collected in Three Top Mountain, North Carolina, USA, to construct a genomic DNA library with 360‐bp inserts. The genomic DNA library was sequenced using an Illumina HiSeq platform at Majorbio (Shanghai, China). Because the original sequencing data have some low‐quality reads, we filtered the original data in order to make the subsequent assembly more accurate. After removing the adapter sequences, we excluded those reads that showed no AGCT sequence at the 5′ end, contained high N content (≥10%), or presented short length (<25 bp) after quality trimming. Finally, we only retained reads with high quality scores; 93.4% of the reads were called accurately when using a threshold of Q20. Using SOAPdenovo version 2.04 splicing software (Luo et al., 2012), the assembly of multiple *k*‐mer parameters was performed on the optimized sequencer, and gaps in the assembly were then locally closed and bases were corrected using GapCloser within SOAPdenovo. A total of 2,464,714 scaffolds were generated. Using MISA (Thiel et al., 2003), a total of 197,407 simple sequence repeat (SSR) loci were detected from the 2,464,714 scaffolds. Primer pairs for the 105 SSR loci with the longest dinucleotide repeats were designed with Primer3 (Rozen and Skaletsky, 1999).

To screen the SSR primers, we conducted PCR amplification using one individual from each of the four populations. PCR amplification was performed in a total volume of 25 μL, containing 12 ng of template DNA, 1.25 units of *Taq* DNA polymerase, 0.8 μM of forward and reverse primer, 0.2 μM of dNTP, 2 mM of MgCl_2_, 2.5 μL of 10× PCR buffer, and 14.25 μL of sterilized double‐distilled water. Thermocycling conditions were 94°C (5 min); followed by 35 cycles of denaturation at 94°C (40 s), annealing at 58°C (45 s), and extension at 72°C (50 s); and a final extension of 72°C (10 min). A total of 105 primers were designed for *A. reclinatum*. The PCR products of 17 primer pairs produced the expected size (Table 1). The remaining 88 primer pairs presented poor amplification. To test the polymorphism level of the 17 primer pairs, the forward primers were labeled using the fluorescent dye FAM. The amplification products were sent to Invitrogen (Shanghai, China) for genotyping. Sequencing Analysis 5.2 (Applied Biosystems, Carlsbad, California, USA) was used to measure the size of the PCR products in the ABI 3730 DNA sequencer (Applied Biosystems). Among a total of 17 primer pairs, five were monomorphic and 12 produced polymorphic sites.

**Table 1:** Characteristics of 12 polymorphic and five monomorphic microsatellite loci developed for *Aconitum reclinatum*

Locus	Primer sequences (5′–3′)	Repeat motif	Allele size range (bp)	*T*_a_ (°C)	GenBank accession no.
AR01^a^	F: TTAGACTTACACGGCCCAGG	(TG)_10_	416–424	60.1	SRR6476459
	R: GTTCCGGGCTTCTCATAACA				
AR02^a^	F: GCTGAACTTGCCATTGTTGA	(CA)_13_	364–374	59.8	SRR6476460
	R: TTCAGCCCTCAGGTCAGTCT				
AR03^a^	F: ATGAATGCAAAGTCCCTTGG	(AG)_10_	397–411	59.9	SRR6476457
	R: GAAGGAGTGCGGTTGATGAT				
AR04^a^	F: TGCTGCTTTCAGGAACAATG	(TC)_10_	288–292	60.0	SRR6476458
	R: AGGAGGACATTGGTGAATCG				
AR05^a^	F: GCTGACAGAGCCATGCTGTA	(GT)_11_	428–438	60.2	SRR6476455
	R: ATGGTATTCCCATGCTCAGG				
AR06^a^	F: CGATCTGACTAGGCCCACAT	(AG)_14_	398–428	60.1	SRR6476456
	R: GGAGAGGGTGGGAATTAGGA				
AR07^a^	F: AACACCCTAGAATCCCCCAC	(TG)_11_	351–367	60.1	SRR6476453
	R: CGACACACACCGAGTGACAT				
AR08^a^	F: ACCCATCATACCAATTCCGA	(AC)_10_	319–325	60.0	SRR6476454
	R: TCACATTGGGAATCAAAGCA				
AR09^a^	F: CGAGCCATTTCACTTGTGTG	(CA)_13_	285–317	60.3	SRR6476451
	R: AGGAGCGAATGTGAGTTGCT				
AR10^a^	F: GAAGGGTATTTTCTCCCCCA	(CA)_11_	292–298	60.1	SRR6476452
	R: ATCCACAGGGACAAACTTGC				
AR11^a^	F: ACCAACTCAGGCATTTGGTC	(GA)_10_	268–282	60.0	SRR6476466
	R: CTCCTCCAATCCCATCAGAA				
AR12^a^	F: ACCGTTTGATCTTGGCAATC	(GA)_13_	258–276	60.0	SRR6476467
	R: TCCTACCCTTGCATCTTTGG				
AR13^b^	F: ACCTAACCGAATTGGCTCCT	(TC)_9_	287	60.3	SRR6476464
	R: GATGTGCATCCCACAATCAA				
AR14^b^	F: TGTTTATACAAGCACCGCGA	(GA)_9_	247	60.0	SRR6476465
	R: AGTACGGACCCTTGATCGTG				
AR15^b^	F: GGAAAGGGATGAGTCGATGA	(GT)_10_	286	59.9	SRR6476462
	R: ACACACACGATTCGGGTACA				
AR16^b^	F: CATCCCACAGACATGAATGC	(CT)_9_	360	60.0	SRR6476463
	R: TGCAAATCACTAGTGCCGAG				
AR17^b^	F: GCTGCATTTGGAAATAGGGA	(TC)_12_	342	59.4	SRR6476461
	R: CCTTCAAACCCAACTCAACC				

Note

*T*_a_ = annealing temperature.

a Tested for polymorphism.

b Monomorphic markers.

Genotypes appeared diploid, displaying at most two alleles per locus per individual. For each locus, the number of alleles per locus (*A*), observed and expected heterozygosity (*H*_o_ and *H*_e_), polymorphism information content (PIC), coefficient of inbreeding (*F*_IS_), null allele frequency (*r*), and Hardy–Weinberg equilibrium were analyzed for the four populations. *A*,* H*_o_, *H*_e_, and Hardy–Weinberg equilibrium were estimated using GenAlEx 6.5 (Peakall and Smouse, 2012). The presence of null alleles was checked using MICRO‐CHECKER 2.2.3 (van Oosterhout et al., 2004). Linkage disequilibrium and *F*_IS_ were estimated using GENEPOP software (Rousset, 2008). CERVUS 3.0.7 was used to calculate PIC and *r* (Kalinowski et al., 2007).

*A* varied from one to seven with an average of 3.48. Levels of *H*_o_ and *H*_e_ ranged from 0 to 1.000 and from 0 to 0.736, respectively, with averages of 0.619 and 0.470. PIC values ranged from 0 to 0.674 with an average of 0.417, and *F*_IS_ values ranged from −1.000 to 1.000 with an average of −0.177. Null allele frequency values ranged from −0.3330 to 0.971 (Table 2).

**Table 2:** Genetic characteristics of the 12 polymorphic microsatellites developed in *Aconitum reclinatum*.^a^

Locus	US15 (*N* = 11)						US17 (*N* = 20)						US22 (*N* = 16)						US27 (*N* = 19)					
	*A*	*H*_o_	*H*_e_ b	PIC	*r*	*F*_IS_	*A*	*H*_o_	*H*_e_ b	PIC	*r*	*F*_IS_	*A*	*H*_o_	*H*_e_ b	PIC	*r*	*F*_IS_	*A*	*H*_o_	*H*_e_ b	PIC	*r*	*F*_IS_
AR01	4	0.909	0.616**	0.539	−0.235	−0.439	5	1.000	0.721***	0.674	−0.175	−0.365	6	1.000	0.613***	0.537	−0.260	−0.611	5	0.895	0.694***	0.645	−0.180	−0.265
AR02	2	0.364	0.397^ns^	0.318	0.044	0.130	4	1.000	0.548***	0.445	−0.304	−0.818	3	0.750	0.656***	0.375	−0.333	−0.111	4	0.895	0.630***	0.558	−0.219	−0.397
AR03	3	0.818	0.533^ns^	0.432	−0.213	−0.500	3	0.750	0.611***	0.531	−0.129	−0.203	3	0.938	0.529**	0.421	−0.315	−0.758	3	1.000	0.547***	0.445	−0.307	−0.819
AR04	3	0.364	0.512*	0.444	0.146	0.333	2	0.350	0.289^ns^	0.247	−0.094	−0.188	2	0.063	0.061^ns^	0.375	—	—	2	0.526	0.488^ns^	0.369	−0.038	−0.053
AR05	2	1.000	0.500***	0.375	−0.333	−1.000	3	1.000	0.524***	0.410	−0.320	−0.905	3	0.938	0.525**	0.421	−0.315	−0.772	6	1.000	0.658***	0.597	−0.228	−0.500
AR06	6	0.909	0.711***	0.665	−0.161	−0.235	3	0.250	0.466*	0.395	0.301	0.484	6	0.688	0.736**	0.586	−0.239	0.098	6	0.474	0.683***	0.647	0.170	0.331
AR07	2	0.000	0.165***	0.152	0.888	1.000	3	0.250	0.501***	0.395	0.290	0.520	2	0.000	0.117***	0.000	—	1.000	2	0.000	0.100***	0.095	0.749	1.000
AR08	2	1.000	0.500***	0.375	−0.333	−1.000	2	1.000	0.500***	0.375	−0.333	−1.000	2	1.000	0.500***	0.375	−0.333	−1.000	4	1.000	0.550***	0.448	−0.302	−0.810
AR09	7	0.545	0.661**	0.635	0.106	0.221	3	0.700	0.516^ns^	0.463	−0.186	−0.333	2	0.063	0.061^ns^	0.375	—	—	5	0.474	0.591***	0.559	0.113	0.225
AR10	3	0.091	0.169***	0.163	0.451	0.500	1	0.000	0.000	0.000	—	—	3	0.063	0.174**	0.375	—	0.659	2	0.000	0.266***	0.231	0.971	1.000
AR11	3	1.000	0.541*	0.436	−0.310	−0.833	6	1.000	0.613***	0.536	−0.261	−0.617	3	1.000	0.529**	0.419	−0.316	−0.882	7	1.000	0.695***	0.645	−0.201	−0.416
AR12	1	0.000	0.000	0.000	—	—	3	0.500	0.551*	0.461	0.030	0.118	5	0.563	0.531^ns^	0.640	—	−0.027	5	0.579	0.457^ns^	0.418	−0.152	−0.241

Note

*A* = number of alleles; *F*_IS_ = coefficient of inbreeding; *H*_e_ = expected heterozygosity; *H*_o_ = observed heterozygosity; HWE = Hardy–Weinberg equilibrium; *N* = number of individuals sampled; PIC = polymorphism information content; *r* = null allele frequency.

a Locality and voucher information are provided in Appendix 1.

b Deviations from HWE using χ^2^ tests: *P ≤ 0.05, **P ≤ 0.01, ***P ≤ 0.001, ns = not significant.

We also tested interspecific transferability of these loci in four additional *Aconitum* taxa (*A. angustius* W. T. Wang, *A. barbatum* Pers., *A. finetianum* Hand.‐Mazz., and *A. sinomontanum* Nakai). Six or seven individuals were selected from each species and used for amplification (Table 3, Appendix 1). A total of six loci (AR01, AR02, AR07, AR08, AR11, AR12) exhibited successful amplifications in *A. finetianum*, five loci (AR01, AR02, AR08, AR11, AR12) in *A. angustius*, and four loci (AR02, AR03, AR08, AR09) in *A. barbatum*. No microsatellite loci were successfully amplified in *A. sinomontanum*.

**Table 3:** Results of cross‐amplification testing showing allele size ranges of microsatellite loci isolated from *Aconitum reclinatum* and tested in four related taxa.^a^

Locus	*A. angustius* (*N* = 6)	*A. finetianum* (*N* = 7)	*A. sinomontanum* (*N* = 6)	*A. barbatum* (*N* = 7)
AR01	418–424	418–424	—	—
AR02	364–374	364–374	—	364–374
AR03	—	—	—	397–411
AR04	—	—	—	—
AR05	—	—	—	—
AR06	—	—	—	—
AR07	—	351–367	—	—
AR08	319–325	319–325	—	319–325
AR09	—	—	—	285–325
AR10	—	—	—	—
AR11	274–282	274–282	—	—
AR12	258–276	258–276	—	—

Note

— = unsuccessful amplification; *N* = number of individuals sampled.

a Locality and voucher information are provided in Appendix 1.

## CONCLUSIONS

A total of 69 alleles were identified for 12 polymorphic loci in the four populations of *A. reclinatum*. These microsatellite loci will be valuable for further interpretation of the fine population structure and conservation strategies of this rare, threatened species.

## DATA ACCESSIBILITY

The raw data has been deposited to the National Center for Biotechnology Information Sequence Read Archive (https://www.ncbi.nlm.nih.gov/sra/SRP129852); the GenBank accession numbers are provided in Table 1. The BioProject ID number is PRJNA427053, and the BioSample accession number is SAMN08218777.

## APPENDIX 1 Voucher and locality information for *Aconitum* species used in this study.

**Table:**

Species	Voucher specimen accession no.^a^	Population location	*N*	Geographical coordinates
*Aconitum reclinatum* A. Gray	US15	Mt. Jefferson, North Carolina, USA	11	36°24′N, 81°27′W
	US17	Mt. Three Top, North Carolina, USA	20	36°25′N, 81°35′W
	US22	Hightown, Virginia, USA	16	38°27′N, 79°42′W
	US27	Mitchell County, North Carolina, USA	19	36°05′N, 82°09′W
*A. angustius* W. T. Wang	LJP195	Shangcheng County, Henan, China	6	31°42′N, 115°31′E
*A. barbatum* Pers.	ZY57	Huairou District, Beijing, China	7	40°57′N, 116°27′E
*A. finetianum* Hand.‐Mazz.	ZY25	Mt. Junfu, Jiangxi, China	7	26°37′N, 115°19′E
*A. sinomontanum* Nakai	ZY46	Kai County, Chongqing, China	6	31°39′N, 108°46′E

Note

*N* = number of individuals sampled.

a One voucher was collected for each sampled population. Herbarium vouchers are deposited in the Herbarium of South China Botanical Garden (IBSC).
